# The relationship between pubertal hormones and brain plasticity: Implications for cognitive training in adolescence

**DOI:** 10.1016/j.dcn.2020.100753

**Published:** 2020-01-22

**Authors:** Corinna Laube, Wouter van den Bos, Yana Fandakova

**Affiliations:** a Center for Lifespan Psychology, Max Planck Institute for Human Development, Berlin, Germany; b Department of Psychology, University of Amsterdam, the Netherlands

**Keywords:** Executive function, Episodic memory, Working memory, Puberty onset, Hormones, MRI

## Abstract

Adolescence may mark a sensitive period for the development of higher-order cognition through *enhanced* plasticity of cortical circuits. At the same time, animal research indicates that pubertal hormones may represent one key mechanism for closing sensitive periods in the associative neocortex, thereby resulting in *decreased* plasticity of cortical circuits in adolescence. In the present review, we set out to solve some of the existing ambiguity and examine how hormonal changes associated with pubertal onset may modulate plasticity in higher-order cognition during adolescence. We build on existing age-comparative cognitive training studies to explore how the potential for change in neural resources and behavioral repertoire differs across age groups. We review animal and human brain imaging studies, which demonstrate a link between brain development, neurochemical mechanisms of plasticity, and pubertal hormones. Overall, the existent literature indicates that pubertal hormones play a pivotal role in regulating the mechanisms of experience-dependent plasticity during adolescence. However, the extent to which hormonal changes associated with pubertal onset increase or decrease brain plasticity may depend on the specific cognitive domain, the sex, and associated brain networks. We discuss implications for future research and suggest that systematical longitudinal assessments of pubertal change together with cognitive training interventions may be a fruitful way toward a better understanding of adolescent plasticity. As the age of pubertal onset is decreasing across developed societies, this may also have important educational and clinical implications, especially with respect to the effects that earlier puberty has on learning.

## 1 Introduction

How does the potential to learn a complex task or acquire a new skill change over the life course? While we never stop learning, there are specific time windows in which the brain may be particularly malleable to new experiences. For example, acquiring a new language is easier in childhood than in adulthood (Mayberry and Lock, 2003). Neuroscientific research has generated a wealth of literature supporting the existence of so-called sensitive phases – limited periods of time, during which the effects of specific experiences on brain structure and function are particularly strong (Hensch, 2005; Hubel and Wiesel, 1963). Typically, periods of increased brain plasticity are thought to occur during early childhood, as the developing brain shows increased malleability in response to different experiences compared to the adult brain (Hensch, 2004). Yet, as cortical regions continue to develop well into young adulthood, sensitive phases for cognitive development may also occur in later childhood and adolescence (Hensch, 2005).

The entry into puberty serves as a clear biological marker of the beginning of adolescence. It is characterized by an increase in gonadal hormone release initiating the development of secondary sexual characteristics, e.g. testicular enlargement in boys and breast development in girls (Shirtcliff et al., 2009). The sex hormones regulating bodily changes are mainly testosterone, estradiol, and dehydroepiandrosterone. Notably, the age of pubertal onset varies considerably among individuals, ranging from 8 to 14.9 years in females and 9.7–14.1 years in males (Lee, 1980).

While it is unlikely that adolescence serves as a sensitive period for earlier-developing processes such as stimulus-response learning or basic sensorimotor processing (Bedny et al., 2018), changes in plasticity in adolescence are most likely for higher-order cognitive functions, such as executive functions and episodic memory, which continue to develop. Currently, there are however different ideas about how plasticity might change as the developing individual transitions from childhood through adolescence into adulthood.

**Fig. 1:**
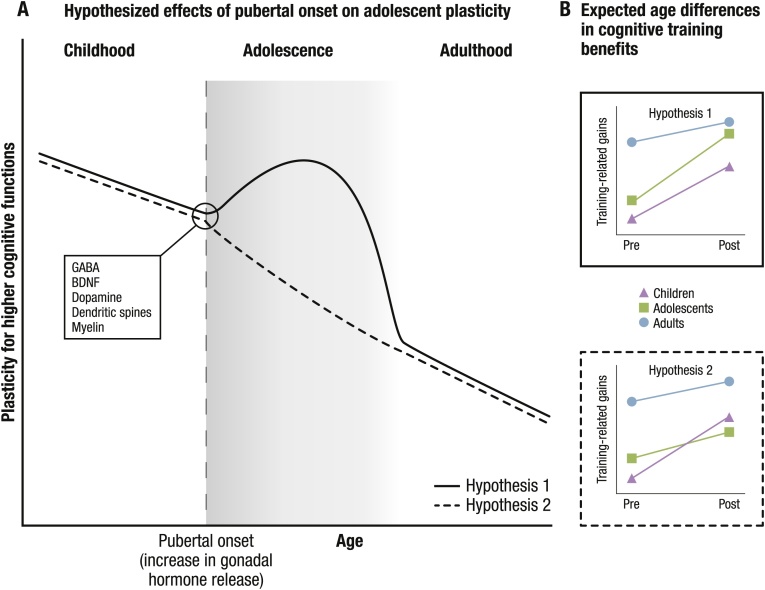
**A**. Hypothesized effects of pubertal onset (increase in gonadal hormone release) on adolescent plasticity, illustrated by two distinct lines. The solid line represents *Hypothesis 1,* stating that plasticity for higher cognitive functions increases after pubertal onset. The dashed line represents *Hypothesis 2,* stating that plasticity for higher cognitive functions decreases after pubertal onset. The box lists potential mechanisms (i.e., neurotransmitters and cell types) that are thought to be involved in the opening or closing of sensitive periods. **B**. Expected age differences in benefits from cognitive training under each hypothesis, separately for pre-pubertal children (triangle and purple), post-pubertal adolescents (square and green), and adults (circle and blue). GABA: γ- aminobutyric acid; BDNF: Brain Derived Neurotrophic Factor.

On the one hand, it has been proposed that adolescence represents a window of increased learning opportunities (Blakemore and Mills, 2014; Steinberg, 2008), and the acquisition of complex social and cognitive skills is thought to be *enhanced* during adolescence (Fig. 1A; Hypothesis 1) (Fuhrmann et al., 2015; Larsen and Luna, 2018). For instance, the reminiscence bump, the finding that autobiographical memories from adolescence and young adulthood are selectively better remembered than experiences before or after this period (Rubin and Schulkind, 1997), may be considered as one example of adolescent-specific plasticity (Fuhrmann et al., 2015). Furthermore, adolescence is thought to represent a second phase of heightened malleability for those social and cognitive functions that rely on the frontoparietal brain network (Steinberg, 2010), which undergoes protracted maturation and continues to change during adolescence (Bunge et al., 2002; Rubia et al., 2006). Together, the temporal co-occurrence of frontoparietal maturation and the observed gains in cognitive abilities suggest the posibility of a sensitive period for higher-order cognitive functions (Fuhrmann et al., 2015; Larsen and Luna, 2018).

On the other hand, plasticity may show a trajectory of overall decrease across the human lifespan that is modulated by alternating periods of plasticity and stability at different points in development (Kühn and Lindenberger, 2016). Accordingly, progression through development is characterized by the sequential closing of sensitive periods for different functional systems and brain regions, which results in generally less potential for change at older ages. The visual system is considered a prime example adhering to this model (Dews and Wiesel, 1970; Werker and Hensch, 2015): If the necessary input is not received during the corresponding sensitive phase, as in the case of visual deprivation during early development, then subsequent environmental changes are not sufficient to resolve developmental deficits (Sharma et al., 2005; Wiesel and Hubel, 2017). Transitioning from childhood to adolescence, it has been suggested that the increase in pubertal hormones represents a key mechanism that contributes to further *decreases* in plasticity (Fig. 1A; Hypothesis 2) (Juraska and Willing, 2017; Piekarski et al., 2017a, Piekarski et al., 2017b). Although there may be relatively more plasticity in adolescence than adulthood, the potential for learning may diminish as children transition into adolescence.

Finally, patterns of plasticity change (i.e., increase, decrease, no change) may not be homogenous but may differ across cognitive domains, reflecting the dynamic interactions between different brain networks involved in these domains, and their specific developmental trajectories. To date, it is unclear which of those hypotheses is favored by empirical evidence. The goal of this review is to explore how the currently available data support the notion of adolescence representing a phase of increased or decreased plasticity, with a focus on pubertal hormones and higher-order cognitive functioning.

First, we provide a working definition of plasticity and discuss how it translates into concrete hypotheses applicable for intervention studies. Second, we review empirical evidence from age-comparative cognitive training studies. Particularly, we explore to what extent these studies show distinct patterns of plasticity across age groups, and whether different domains of cognitive functioning may show evidence for increased or decreased plasticity in adolescence. Third, we review animal literature suggesting that pubertal hormones can influence the neural mechanisms involved in the regulation of sensitive phases of development. In addition, we discuss human imaging studies exploring the impact of pubertal hormones on changes in brain function and structure. Finally, we discuss the resulting implications for learning in adolescence and present a roadmap on how to study the interacting effects on maturation and pubertal development on plasticity.

## 2 Plasticity as the capacity for change in brain and behavior

Broadly speaking, the concept of plasticity refers to any type of change in brain and behavior (see Kadosh et al., 2013; Lundborg, 1998). Lövdén et al., 2010 proposed a theoretical framework of plasticity that operationally defines the term as a secondary response to an initial change in the system. That is, plastic changes in brain and behavior are thought to result from experience and are therefore distinct from changes caused by normative maturation. Plasticity in this framework is defined as the system’s ability to change, or *potential for change,* and is metabolically costly. Plastic changes are thus likely to occur only when there is a prolonged mismatch between the currently available resources and environmental demands (Lövdén et al., 2010;Lövdén et al., 2013 Cognitive training studies represent one way to increase environmental demands on specific cognitive processes in order to examine behavioral and neural manifestations of plasticity. Yet, improvements in cognitive functioning can also be observed in the absence of changes in the range of available resources. Based on the framework by Lövdén et al. (2010), such improvements would correspond to greater *flexibility*, which represents the ability to optimize available resources and behaviors in order to rapidly adapt to current environmental demands. Thus, plasticity *changes* existing neural resources, whereas flexibility *uses* them (Kühn and Lindenberger, 2016).

Therefore, it is important to distinguish between normative, maturation-related processes and plasticity-related processes resulting from experience (Galván, 2010). Based on the framework by Lövdén et al. (2010), improvements in cognitive functioning across childhood and adolescence may reflect the interaction between maturation-dependent changes in the system (leading to greater available resources per se) along with changes due to a mismatch between environmental demands and available resources, reflecting manifestations of experience-dependent plasticity. In turn, individual differences in maturation and resulting available resources are likely to influence the likelihood for plastic changes with learning (Galván, 2010) such that a similar environmental challenge may or may not induce plastic changes in brain structure depending on the individual’s currently available resources (Lövdén et al., 2010).

To conclude, plasticity denotes the brain’s capacity to respond to changing environmental demands with structural brain changes, resulting in lasting behavioral alterations. This potential for change with learning may critically interact with normative brain maturation, leading to increased or decreased plasticity in adolescence. In order to test these assumptions, we next formulate concrete hypotheses to guide our literature review.

## 3 Consequences of increased versus decreased plasticity for cognitive training in adolescence

Based on the theoretical framework of plasticity proposed by Lövdén et al. (2010), what would increased or decreased plasticity during adolescence concretely translate to? Imagine a study in which participants of different age groups such as pre-pubertal children, post-pubertal adolescents, and adults receive a cognitive training intervention. If adolescence represents a phase of *increased plasticity* for higher-order cognition, one would expect adolescents to show greater benefits from training compared to children (see Fig. 1A Hypothesis 1 and Fig. 1B upper panel). In addition, while the transition to adolescence may mark a general change in potential for change relative to childhood, a specific sensitive period would be characterized by differences as compared also to adults (Bedny et al., 2018; Fuhrman et al., 2015; see Fig. 1A Hypothesis 1). In contrast, if *plasticity decreases* in adolescence, for example due to puberty-related hormonal changes, we would expect adolescents to benefit less from the intervention than children (see Fig. 1A, Hypothesis 2 and Fig. 1B lower panel). Put differently, if adolescence is marked by decreased plasticity for higher-order cognitive functioning, the training intervention would be expected to be more effective in children than in adolescents. At the same time, adolescents would be expected to either show comparable or possibly even higher training benefits than adults. As higher-order cognition relies on complex interactions among different cortical and subcortical networks, which vary in their developmental trajectories, plasticity may also manifest differently across different cognitive domains.

We assume that if there are any adolescence-specific changes in plasticity, they are more likely for higher-order cognitive functions that continue to develop in adolescence (see also Fuhrmann et al., 2015). Extensive research has demonstrated that executive functions, defined as higher-order cognitive control of thought, action, and emotion (Zelazo et al., 2008), continue to develop well into adolescence and early adulthood (Luna et al., 2004, 2015; Peper and Dahl, 2013; Satterthwaite et al., 2013). Specifically, *updating* (i.e., the rapid addition, deletion, and ongoing monitoring of working memory contents), *inhibition* (i.e, the deliberate overriding of prepotent responses), and *shifting* (i.e., the flexible switching between tasks or mental sets) (Miyake and Friedman, 2012), continue to improve during the first two decades of life (Brydges et al., 2014; Huizinga et al., 2006). Executive functions critically depend on the lateral frontoparietal brain network (Brass and von Cramon, 2004; Bunge et al., 2002), which shows protracted development in terms of structure, function, and connectivity both within the network and with other networks (Crone and Dahl, 2012). The prefrontal cortex (PFC) is among the regions that show the latest maturation, with gray matter volume increasing until middle childhood followed by subsequent volume decreases during adolescence (Giedd et al., 1999; Sowell et al., 2003). White matter connections between frontal and parietal regions, such as the superior longitudinal fasciculus, also increase nonlinearly across adolescence and are particularly pronounced in early adolescence, between 10 and 15 years of age (e.g., Lebel and Beaulieu, 2011). Adolescence is also marked by the integration of association and projection fibers that connect the PFC with subcortical regions such as the striatum and the hippocampus (e.g., Asato et al., 2010). In addition, episodic memory, the ability to remember events situated in particular times and places in the past, also continues to improve throughout late childhood and adolescence (Schneider and Pressley, 1997; Fandakova et al., 2017). These improvements have been related to increased efficiency of PFC-based control processes that guide and enhance memory encoding and retrieval (Fandakova et al., 2018) along with changes in hippocampus (Keresztes et al., 2017) and PFC–hippocampus connectivity (Murty et al., 2016).

In sum, executive functions and episodic memory are domains with protracted development during adolescence. Thus, we expect that changes in plasticity during adolescence are likely to occur in those domains. In order to explore the extent to which plasticity is decreased or increased for these different cognitive domains, we next review age-comparative intervention studies that trained executive functions or episodic memory (see Table 1). Here, we were particularly interested in comparisons of training-related benefits between different age groups (children, adolescents, and adults) that help to infer whether and if so, for which higher-order cognitive functions adolescence is associated with increased or decreased plasticity. Note that we review the studies with their original age group labels, but provide concrete age ranges to enable the comparison of results across studies.

**Table 1:** Overview of age-comparative cognitive training studies.

Authors	Age groups	Domain	Training design	Training task	Results
Brehmer et al. (2007; 2008)	younger children (9–10 years, *N* = 23), older children (11–12 years, *N* = 27), young adults (20–25 years, *N* = 29), older adults (65–78 years, *N* = 29)	Episodic memory	two to six individualized training sessions (until asymptotic level of performance) with pre-post test; follow-up after 11 months*	Participants learned to encode and retrieve lists of words using an imagery-based mnemonic strategy (Method of Loci)	older adults < younger children < older children < young adults
Brehmer et al. (2016)	children (10-11years, *N* = 28), young adults (21–25 years, *N* = 31), older adults (63–70 years, *N* = 22)	Episodic memory	four separate training sessions with pre-post test	Participants learned concrete and unrelated German noun pairs using a visual imagery strategy	children = young adults = older adults
Shing et al. (2008)	children (10–12 years, *N* = 43), adolescents, (13–15 years, *N* = 43), young adults (20–25 years, *N* = 42), older adults (70–75 years, *N* = 42)	Episodic memory	five separate training sessions as follow-up experiment with pre-post test (where pre-test was post-stategy of previous experiment 4.5 months earlier)	Participants learned German-Malay word pairs by using an imagery strategy	children = adolescents > adults
Jolles et al. (2012)	children (11–13 years, *N* = 10), young adults (19–25 years, *N* = 15)	Working memory	six week training with pre-post test of changes in behavior and task-related neural activity measured by fMRI	Participants learned sequences of objects and indicated their positions within each sequence (n-back task)	children = young adults in performance no age differences in task-related activation within frontoparietal network
Jolles et al. (2013)	children (11–13 years, *N* = 9), young adults (19–25 years, *N* = 15)	Working memory	six week training with pre-post test of changes in behavior and resting-state functional connectivity measured by fMRI	Participants learned sequences of objects and indicated their positions within each sequence (n-back task)	children = young adults in performance changes in resting-state connectivity in young adults, but not in children
Cepeda et al. (2001)	younger children (7–9 years, *N* = 14), older children (10–12 years, *N* = 12), adolescents (13–20 years, *N* = 17), young adults (21–82 years, *N* = 109)	Task-set shifting	two sessions consisting a computerized task-switching paradigm within a period of one week	Participants saw different digits and had to name either the number or the amount of numbers displayed on the screen	children > adolescents & adults
Karbach and Kray (2009)	children (8–10 years, *N* = 56), young adults (18–26 years, *N* = 56), older adults (62–76 years, *N* = 56)	Task-set shifting	four training sessions with pre-post test over 6–8 weeks	Participants shifted between two different task sets every two traials.	children > young adults
Knoll et al. (2016)	younger adolescents (11–13 years, *N* = 57), midadolescents (13–16 years, *N* = 57), older adolescents (16–18 years, *N* = 60), young adults (18–33 years, *N* = 36)	Relational reasoning, numeriosity discrimination, & face perception	20 days of online training; pre-post test; follow-up after 3–9 months*	*Relational reasoning*: Modified Raven‘s Progressive Matrices *Face perception task:* decide if two faces same or different; *Numeriosity discrimination*: Compare two different sets of colored dots that vary in number	younger adolescents < older adolescents

Note: Age group labels for specific age spans are used differently across studies. For results, comparisons of training benefits are depicted. Asterisk indicates an adaptive training design.

Due to lack of age-comparative training studies in inhibition, we only discuss inhibition in the context of other cognitive functions.

## 4 Cognitive training studies comparing different age groups

### 4.1 Episodic memory

While newborns already show signs of the ability to retain information about the past, episodic memory continues to develop throughout childhood and adolescence (Ghetti and Bunge, 2012). One fundamental mechanism underlying memory development is the binding of different features of an event, such as when, where, or how it happened, which is supported by the hippocampus and its corresponding subregions (Keresztes et al., 2017). While the ability to remember single items reaches adult-like levels by early childhood (e.g., 4–6 years; e.g., Sluzenski et al., 2006), different types of memory binding show distinct developmental trajectories. For example, while item–location associations are remembered at levels comparable to adults by about 10.5 years, item–time and arbitrary item–item associations reach adult-like levels at only around 12–12.5 years of age, and are closely related to hippocampal maturation (Lee et al., 2019). In addition, the ability to efficiently guide and control memory encoding and retrieval continues to improve over development. Children become better at using elaborative encoding strategies, especially between preschool years and adolescence (Schneider and Pressley, 1997). The ability to monitor and make decisions about memory accuracy also increases well into adolescent years due to protracted PFC maturation (Fandakova et al., 2017).

Brehmer et al. (2007) examined episodic memory plasticity from middle childhood to old age in a multi–session training study. An imagery-based mnemonic strategy was taught and practiced in four different age groups: younger children (9–10 years), older children (11–12 years), younger adults (20–25 years) and older adults (65–78 years). Specifically, participants learned a mnemonic strategy, in which they encoded and retrieved words by location cues. The training was adaptive, in which the number of practice sessions ranged from two to six, depending on when an asymptotic level of performance was reached. Immediately after training, younger adults showed the largest gains in memory performance, followed by both child groups who in turn benefited more from practicing the strategy compared to older adults. A follow-up session 11 months later showed a similar pattern of results, with higher performance in young adults than in both child groups, indicating that training-related benefits were maintained across all age groups (Brehmer et al., 2008). Taken together, both 9–10-year-olds and 11–12-year-olds benefited less from training than younger adults, but more than older adults. These results suggest that the 11–12-year-old children transitioning into adolescence did not necessarily show higher plasticity in memory for item–location pairs compared to the 9–10-year-olds or young adults.

Similarly, Shing et al. (2008) trained episodic memory in four age groups: children (10–12 years), adolescents (13–15 years), younger adults (20–25 years) and older adults (70–75 years). Participants learned pairs of German–Malay words using a keyword imagery strategy. The strategy entailed finding a meaningful connection for the unfamiliar Malay word and integrating it with the familiar German word through imagery. A follow-up four and a half months later examined further improvements associated with practice across five sessions. Results revealed that the 10–12-year-olds and 13–15-year-olds benefited significantly more from the strategy instruction and from practicing the keyword imagery strategy than did younger and older adults. However, children and adolescents did not differ in training benefits, suggesting similar levels of memory plasticity between these age groups.

Finally, Brehmer et al. (2016) also trained mnemonic skills in three age groups, including children (10–11 years), young adults (21–25 years), and older adults (63–70 years) across four separate training sessions. Here, participants learned associations between unrelated German nouns using a visual imagery strategy. In contrast to Shing et al. (2008) and Brehmer et al., 2007, Brehmer et al., 2008, all age groups improved with practice from pre- to post-test, and there were no differences in training benefits across different age groups.

In sum, the available evidence in episodic memory suggests that adolescents do not appear to show specific benefits of training compared to children or adults. It should however be noted that even the younger groups in these studies for the most part fall within the range of adolescence regarding age, thereby preventing clear conclusions about differences between children and adolescents in the potential to change episodic memory ability with training.

### 4.2 Working memory

Working memory, the capacity to maintain and manipulate information in the service of goal-directed behavior, increases across childhood and adolescence (Sander et al., 2012) with pronounced improvements well into young adulthood (Finn et al., 2010; Satterthwaite et al., 2013). These improvements have been associated with increased connectivity and task-related engagement in parietal and PFC regions (Crone et al., 2006; Ullman et al., 2014). Moreover, striatum activity has been shown to predict future working memory performance (Darki and Klingberg, 2015), indicating the critical role of this structure for working memory development.

Jolles et al. (2012) conducted a working memory training study with an n-back task in children (11–13 years) and young adults (19–25 years) over the course of six weeks. Participants had to learn sequences of objects and eventually indicate their positions within each sequence. Following training, 11–13-year-olds reached adult-like performance levels, indicating higher training-related benefits than adults. However, adults already reached ceiling levels of working memory performance early on during training, leaving no room for improvement and making it difficult to directly compare their training benefits to the benefits of children. In addition, Jolles et al. (2012) investigated neural changes associated with the working memory training and found that 11–13-year-olds showed increased frontoparietal activation during working memory maintenance after practice such that their patterns of activation became more similar to adults. Age-related differences in neural activation within the fronto-parietal network were thus considerably reduced after practice. Taken together, this study demonstrated that working memory training reduced behavioral as well as neural differences between 11–13-year-old children and adults. Yet practice-related changes in neural activation were not tested in the adult group, and thus the extent to which these changes differed from children is unclear.

In a follow-up study, Jolles et al. (2013) carried out the same working memory training in 12-year-old children and young adults (19–25 years) and found similar results. While the 12-year-olds showed lower working memory capacity prior to training, there were no significant differences between children and adults after training. At the same time, practice-related changes in fronto-parietal and default network’s resting-state connectivity were only apparent in young adults, but not in children (Jolles et al., 2013). These results suggest that, at least in terms of changes in resting-state connectivity, young adults may be more malleable than children.

In sum, the findings from these two studies contrast each other. Potential reasons for these differences may include distinct analysis approaches (task-related activity versus functional connectivity), as well as different study phases being examined (during task execution versus rest periods after task execution).

### 4.3 Task-set shifting

Task-set shifting refers to the ability to flexibly switch attention between different sets of tasks, rules, or features. Children can switch between simple task sets by around age 5 (Buttelmann and Karbach, 2017). However, task-set shifting continues to improve through adolescence (Best and Miller, 2010; Karbach and Unger, 2014), with different cognitive control components developing at different rates. Here, switch costs, which are thought to reflect the need to resolve interference from no-longer relevant task sets and to reconfigure a newly relevant task set, decrease over development and reach adult-like levels by around age 11. In contrast, mixing costs, which indicate the maintenance and coordination of multiple task sets, continue to improve well into adolescence and reach adult levels around age 15–17 (Crone et al., 2004; Huizinga and van der Molen, 2007; Reimers and Maylor, 2005). On the neural level, frontoparietal regions become more selectively engaged in task-set shifting with age (Church et al., 2017; Crone et al., 2006).

Cepeda et al. (2001) examined changes in the ability to switch between different task sets across two practice sessions. Specifically, 152 individuals ranging from 7 to 82 years of age saw different digits and had to name either the number or the amount of numbers displayed on the screen depending on a cue displayed simultaneously with the numbers. Results revealed that while switch costs decreased in all age groups with practice, 10–12 year-olds showed the largest improvements. It has to be noted that 7–9 year-olds were not included in this analysis due to a lack of data for the second session. In addition, 13–20-year-olds were grouped together, which prevented the examination of adolescence-specific effects. Yet, these results suggest that 10–12-year-olds have larger training-related benefits compared to (older) adolescents and adults.

In another study, Karbach and Kray (2009) trained children (8–10 years), young adults (18–26 years) and older adults (62–76 years) in task-set shifting using the same or variable tasks across four training sessions. For example, in one task, subjects had to indicate whether pictures showed planes or cars and whether one or two vehicles were presented. The results showed decreases in switch and mixing costs across groups over time. Summarizing across different training manipulations, training-related decreases in both types of costs were most pronounced in 8–10-year-olds when they trained on the same task repeatedly. In addition, transfer to structurally similar tasks that were not used during training was more pronounced in children and older adults than in younger adults. The findings of Karbach and Kray (2009) therefore suggest that children benefit more from a task-set shifting training compared to young adults (see also Karbach et al., 2017).

Taken together, the results of these studies indicate that task-switching may be more malleable in childhood than in young adulthood. However, these studies do not clarify whether the potential for change in task-switching ability differs in adolescence specifically.

### 4.4 Relational reasoning

Relational reasoning, or the ability to consider relations among representations that go beyond stimuli features, improves during childhood and well into adolescence (Vendetti and Bunge, 2014). On the neural level, patterns of activation in PFC and parietal regions become similar to adults only in late adolescence (Dumontheil, 2014; Wendelken et al., 2011). Accordingly, increasing structural connectivity between PFC and parietal regions has been found to predict future changes in reasoning ability in a large longitudinal sample between 6 and 22 years (Wendelken et al., 2017).

Knoll et al. (2016) trained four different age groups, younger adolescents (11–13 years), mid-adolescents (13–15 years), older adolescents (15–18 years), and adults (18–33 years) on adaptive numerosity discrimination, relational reasoning, and face perception over the course of 20 days of online training. In addition, they also included a follow-up session three to nine months after training. Post-tests immediately after training indicated that 15–18-year-olds and adults benefited most from the training in relational reasoning and in numerosity discrimination. Improvements in relational reasoning were sustained for all age groups in the follow-up session, whereas maintained benefits in numerosity discrimination were only found for adults. There were no age differences for the face-perception training. Based on these results, training complex cognitive skills such as relational reasoning seems to result in greater performance benefits during later adolescence than during early adolescence. Nevertheless, this study does not make it possible to gauge whether adolescence represents a specific window of plasticity, as none of the participants were younger than 11 years. Thus, these results do not necessarily contradict the findings discussed above, showing that children benefit more from training than young adults across different domains (Jolles et al., 2012; Karbach and Kray, 2009; Karbach et al., 2017; Shing et al., 2008). In contrast, some of the reviewed studies also found greater plasticity in young adults compared to children on both the behavioral and the neuronal level (Brehmer et al., 2007; Jolles et al., 2013), while one study did not find any age differences (Brehmer et al., 2016).

There are multiple plausible explanations for these different patterns of results across studies. On the one hand, they may reflect differences in plasticity across different cognitive domains. This idea would be consistent with the different ages at which children reach adult-like levels of task performance across domains. For example, the fact that older adolescents benefited most from practicing relational reasoning may reflect the protracted maturation of this ability relative to episodic memory or task-set shifting (cf. Larsen and Luna, 2018). Alternatively, the different patterns of results may be related to the specific training regimes implemented across studies, such as practicing tasks versus strategy instruction, or whether the difficulty of the practice problems was adapted to the individual participants.

In sum, the current evidence for adolescent-specific plasticity changes for cognitive training is mixed. In particular, due to the different ages and splits of age groups, we cannot draw general conclusions on whether and which cognitive training interventions show higher or lower effectiveness in adolescence at this point. Furthermore, the absence of pubertal measures in existing studies does not allow us to examine the relation between pubertal hormones and changes in brain plasticity. To illustrate potential consequences of the effects of pubertal hormones on plasticity, we next review what is known about the ways in which mechanisms of plasticity are impacted by pubertal hormones in animals.

## 5 Effects of pubertal hormones on neurobiological mechanisms of plasticity

If puberty contributes to the regulation of sensitive periods for cognitive development, sex steroids such as testosterone and estradiol, which are both elevated during puberty (Shirtcliff et al., 2009), may play an important role in regulating the mechanisms of plasticity. Specifically, pubertal development initially starts with the release of dehydroepiandrosterone (DHEA) and its sulfate (DHEA-S) by the adrenal glands, which are responsible for the development of pubic hair and changes in body odor and skin. This process, called adrenarche, usually occurs between the ages of 6 and 9 years, and earlier in girls than in boys (Biro et al., 2014; Patton and Viner, 2007;). Gonadarche usually starts about two years after adrenarche. It is characterized by a rapid increase in testosterone and estrogen levels released by the testes and ovaries, respectively, initiating the development of secondary sexual characteristics such as testicular enlargement in boys and breast development in girls (Shirtcliff et al., 2009). As the increase of DHEA and DHEA-S are slower and more prolonged (up to the early 20s) compared to the relatively steeper increase in testosterone and estrogen during gonadarche, visible puberty-typical changes usually occur and accelerate during gonadarche, which is also thought to better capture pubertal onset.

We now turn to neurochemical mechanisms of plasticity, that are thought to be involved in the regulation of sensitive periods. In particular, we focus on those thought to open and close sensitive periods (Hensch, 2005). These mechanisms include various changes in neurotransmitter levels, receptors and signaling, as well as in gene expression.

### 5.1 γ-Aminobutyric acid (GABA)

Generally speaking, the opening of sensitive periods is thought to be driven by inhibitory neurotransmission that plays a key role in regulating the balance of excitation and inhibition in the brain (Dorrn et al., 2010). At the beginning of a sensitive phase, inhibitory interneurons, particularly parvalbumin-positive large basket (PV) cells, increase and mature due to environmental input such as sensory stimulation (Hensch, 2005). These inhibitory interneurons lead to a suppression of spontaneous neural activity in favor of stimulus-evoked activity which in turn shifts the balance between inhibitory and excitatory firing, resulting in the establishment of more powerful and fine-tuned information flow (Fagiolini and Hensch, 2000; Toyoizumi et al., 2013). PV cells operate via GABA-ergic neurotransmission, such that an increase in GABA is thought to be related to the onset of a sensitive period.

Increases in specific subunits of GABA receptors, namely GABA A, and in PV cells contribute to the opening of sensitive periods and coincide with the onset of puberty (Shen et al., 2007). For example, Wu et al. (2014) examined protein expression of PV cells and the GABA synthesizing enzyme GAD67 in the hippocampus during adolescence, specifically focusing on their interaction with steroid hormones. For female mice, ovariectomy around puberty onset significantly reduced PV expression in the dorsal hippocampus, while for male mice adolescent castration and treatment with testosterone had no effect on PV and GAD67 enzyme expression. Since expression levels of PV cells have also been related to memory consolidation and retrieval in the adult hippocampus (Donato et al., 2013), the results of this study suggest that pubertal hormones show sex- and region-specific effects on plasticity, where estradiol may be necessary for initiating a sensitive period for memory improvement in females via changes in protein and enzyme expression of PV cells and GABA in the dorsal hippocampus.

In another study, Shen et al. (2010) showed that GABA A receptors increased at pubertal onset in the mouse hippocampus and affected activation of *N*-methyl-d-aspartate (NMDA) receptors, which are essential for learning and memory (Nakazawa et al., 2004). Specifically, in this study increases of GABA receptors in the hippocampus at puberty onset led to decreases in NMDA receptor activation, resulting in impaired induction of long-term potentiation (LTP) via inhibition of GABA receptors. As a consequence, pubertal mice failed to learn a behavioral spatial task dependent on LTP in the hippocampus. These results suggest that pubertal onset may decrease plasticity specifically in the hippocampus via changes in GABA receptor density. The negative effect of pubertal hormones on hippocampal plasticity is indirectly further supported by findings that testosterone implants given to gonadectomized rats during puberty significantly decreased plasticity of the hippocampal subfield CA1 in adulthood (Harley et al., 2000). Similarly, a study by Hebbard et al. (2003) also found that pubertal testosterone was related to LTP in the hippocampus in male rats, along with reduced social memory. In sum, the onset of puberty in rodents is associated with increases in GABA receptors with possible negative effects on hippocampal-based learning and plasticity.

A recent study by Piekarski et al. (2017a) combined the manipulation of pubertal hormones with whole-cell recordings of excitatory and inhibitory inputs onto pyramidal cells in the cingulate cortex and somatosensory barrel cortex of the mouse. They found that pre-pubertal, but not post-pubertal ovariectomy blocked an increase in inhibitory neurotransmission specifically in the cingulate cortex, but not in somatosensory areas. Moreover, pre-pubertal hormone treatment with estradiol and progesterone accelerated the maturation of inhibitory neurotransmission, such that hormone-treated mice exhibited stronger synaptic depression at inhibitory synapses, suggesting greater GABA release. Together, these results are consistent with the findings above that manipulations of pubertal onset are associated with changes in inhibitory neurotransmission. These findings were specific to the frontal cortex, suggesting that the effects of hormones are regionally focused. Notably, early puberty onset via pre-pubertal hormone treatment had a negative impact on behavioral flexibility such that mice with peri-pubertal exposure to gonadal hormones required more trials to reach criterion performance during the reversal phase of a reversal-learning task compared to controls. At the same time, ovariectomy did not result in learning deficits when tested in adulthood. These results suggest that speeding up pubertal onset may have negative effects on learning, but at the same time the maturation of reversal learning does not depend exclusively on hormones.

According to animal studies, puberty onset is, in sum, closely related to GABA and results in increased inhibitory neurotransmission. The effects of puberty onset on inhibitory neurotransmission are not uniform across the brain, but are region-specific and may also depend on sex and pubertal timing. For instance, while a normative rise in estradiol may increase plasticity in the hippocampus, an earlier puberty onset may decrease plasticity in the frontal cortex. Furthermore, the mapping of specific puberty-related changes in neural plasticity to concrete behavior is complex and may depend on the corresponding functional domain. For example, when explicitly tested in behavioral tasks, puberty-related increases in inhibitory neurotransmission were found to be associated with decreased learning benefits, suggesting that behavioral manifestations of plasticity may be decreased after entry into puberty.

### 5.2 Brain derived neurotrophic factor (BDNF)

BDNF is synthesized by pyramidal neurons and plays an important role in the differentiation and maturation of interneurons (Glorioso et al., 2006). It is necessary for regulating the onset of critical periods by promoting GABAergic neuron development, including neuron density and GAD67 gene expression, as well as levels of parvalbumin (Arango-González et al., 2009; Glorioso et al., 2006; Villuendas et al., 2001). In addition, both BDNF expression and GABA transmission have been shown to be reduced in sensory deprivation experiments that delay the onset of a sensitive period (Hensch, 2005; Morales et al., 2002), indicating that increased BDNF may be related to the opening of sensitive periods.

With respect to the relation between pubertal onset and BDNF, Hill et al. (2012) measured serum blood levels of testosterone and estradiol weekly from pre-pubescence to adulthood in male and female mice. Additional analyses included BDNF and tyrosine kinase (Trk) B, a BDNF receptor, in cortico-striatal and hippocampal regions. In male mice, a peak in testosterone at pubertal onset was positively correlated with BDNF expression in striatal and frontal regions. BDNF expression subsequently decreased as levels of testosterone dropped shortly after puberty onset. In contrast, TrkB expression was negatively correlated with testosterone in striatal and frontal regions, but not in the hippocampus. In female mice, there were no significant correlations between serum estradiol and BDNF-TrkB expression or signaling during adolescence. Taken together, similar to GABA, pubertal hormones show sex- and region-specific effects depending on the specific mechanisms including BDNF expression and BDNF-TrkB signaling: whereas testosterone increased BDNF expression in males, it was negatively correlated with BDNF-TrkB signaling. Similarly, a study by Purves-Tyson et al. (2015) found that testosterone removal by gonadectomy in male monkeys and rats shortly before puberty onset increased gene expression of specific BDNF transcripts, whereas testosterone replacement before puberty onset prevented these increases in BDNF mRNA in frontal cortices. Another study by Liu et al. (2012) found that BDNF signaling regulated by sex hormones directed pruning of sensory axons in the mammary gland. As such, developmental changes in BDNF in the mammalian cortex may be related to pubertally driven changes in gonadal hormones as part of a normative closing of a window of increased plasticity in the frontal cortex (Purves-Tyson et al., 2015).

### 5.3 Dendritic spines

Changes in dendritic spines are thought to be related to plasticity such that less density is associated with more efficient connectivity and a potentially more stable system that is less prone to change (Dahl et al., 2018).

With respect to a relation between dendritic spines and puberty onset, a study by Meyer et al. (1978) found that castration of rats before puberty prevented a peak in dendritic spine formation in the hippocampus around puberty onset. More specifically, gonadectomized rats showed no change in the number of dendritic spines over the course of adolescence, while a control group showed a peak in dendrites around puberty onset. This peak in dendritic spines in the control group was followed by a decrease in spine density after puberty until similar numbers of dendrites as the experimental group were eventually reached in young adulthood. Pre-pubertal castration has also been shown to decrease the number of dendritic spines in the medial amygdala in adult mice compared to a control group (Cooke and Woolley, 2009). Finally, estrogens have been shown to increase dendritic spine density in pyramidal cells in CA1 of the hippocampus in female rodents, but not in males (Leranth et al., 2003; MacLusky et al., 2005).

Together, these findings, mainly from rodent studies, suggest that hormonal increases at pubertal onset promote dendritic spine density in hippocampus and amygdala, potentially enhancing plasticity in these areas.

### 5.4 Myelination

Myelination is considered a braking factor of plasticity, as it may prevent further structural changes (Werker and Hensch, 2015). A study by Yates and Juraska (2008) tested how the number of myelinated axons in young adulthood were affected by ovariectomy in the rat corpus callosum. They found that ovariectomized animals had a greater number of myelinated axons in the splenium compared to intact animals, while total axon number was not affected. This suggests that estradiol may decrease myelination and thus enhance plasticity. This idea is in contrast to a study by Prayer et al. (1997) that found that peri-pubertally estrogen-treated rats showed accelerated white matter maturation across development in the anterior and hippocampal commissure, while peri-pubertally testosterone-treated rats showed the opposite effect.

In sum, pubertal hormones have been shown to both decrease and accelerate myelination in a sex- and region-specific manner and may thus promote both directions of plasticity change after pubertal onset.

### 5.5 Dopamine

Dopamine, a neurotransmitter critical for reward-based learning (Wise, 2004), is often discussed in relation to adolescence (Larsen and Luna, 2018). But does it play a role in regulating plasticity during adolescence in relation to puberty? Dopamine has been suggested to increase neural sprouting and synaptogenesis (Stroemer et al., 1998). Dopamine receptors (i.e., the D1 receptor) are particularly important for modulating synaptic plasticity in frontal regions (Selemon, 2013). In rodents, there are pronounced increases in dopaminergic innervation of PFC over the course of adolescence (Naneix et al., 2012). Pubertal hormones have been shown to positively impact dopamine synthesis and signaling in a region-specific manner (Laube and van den Bos, 2016), resulting in increased reward-motivated behaviors and facilitated learning. The timely co-occurrence of dopamine increases with pubertal onset may be crucial for adolescents’ exploratory behavior (Kuhn et al., 2010), which in turn may promote experiences that initiate a sensitive period for learning during adolescence (Larsen and Luna, 2018). Based on the framework of Lövdén et al. (2010), dopamine may increase flexibility by making adolescents more sensitive to novel situations, which they then have to solve independently, thus increasing their behavioral repertoire of available knowledge and strategies. Additionally, increased dopamine signaling associated with puberty onset may interfere with synaptic pruning mechanisms, increasing the malleability of frontal regions, especially in situations with higher reward-circuitry activation. A recent study with mice directly tested whether adolescence is marked by a period of increased sensitivity to change in mesocortical dopamine innervation and inhibitory behavior (Reynolds et al., 2019). Dopamine development was disrupted in early-adolescent, mid-adolescent, or adult animals via exposure to amphetamine, which alters mesocortical dopamine connectivity. This treatment led to impaired performance on an inhibition task and to reduced PFC dopamine turnover in adulthood, crucially only in the early-adolescent exposure group. This may be due to the negative effects of amphetamine on the guidance cue receptor DCC, which has been associated with regulating dopamine axonal growth (Reynolds et al., 2018). While the potential effects of gonadal hormones on the regulation of DCC receptor expression are topics for future research (Hoops and Flores, 2017), these results point to an instrumental role of dopamine for increased plasticity in cognitive control specifically in early adolescence.

## 6 Interim summary

Taken together, the findings reviewed above suggest that pubertal hormones, which drastically increase at pubertal onset, may indeed play a role in regulating plasticity. Pubertal onset modulates neurochemicals and cell properties implicated in the opening of sensitive periods, such as the maturation of PV cells, as well as increases in GABA and BDNF (Werker and Hensch, 2015, summarized in Fig. 1A). This impact of pubertal hormones may, however, be more nuanced than absolute, associated with both increases and decreases in plasticity depending on the specific region or neural circuit, and interacting with sex, as well as with the timing of puberty onset. That is, whereas a normative increase in estradiol seems to increase plasticity via changes in expression of GABA and PV cells in the hippocampus (Wu et al., 2014), as well as by decreasing myelination (Yates and Juraska, 2008), an earlier exposure to estradiol initiating early pubertal timing may be related to decreased plasticity (Piekarski et al., 2017a; Prayer et al., 1997). Studies including behavioral correlates of learning showed a general trend towards a drop in performance after pubertal onset, suggesting a decrease in learning (Piekarski et al., 2017a; Reynolds et al., 2019). These results are consistent with recent findings from skill learning showing that adolescent gerbils demonstrated attenuated auditory discrimination learning and decreased auditory-cortex sensitivity during training compared to both juvenile and adult animals (Caras and Sanes, 2019). The extent to which these declines in skill acquisition represent a puberty-related decrease in neural plasticity and/or reflect reorganization of the available behavioral repertoire is currently unclear.

As most of the research to date has been conducted in animals, it is clearly difficult to apply the reported findings directly to human development. To gain more insight into how pubertal changes in gonadal hormones impact on neuroplasticity in humans, we next review longitudinal imaging studies conducted in humans that focused on pubertal development.

## 7 Effects of pubertal hormones on change in brain structure and function

Only a few studies have investigated the relationship between pubertal hormones and structure and function in the human brain. Evidence from cross-sectional studies is mixed: Some studies find an association between gonadal hormone levels and gray matter in frontal areas (Bramen et al., 2012; Koolschijn et al., 2014) or functional activity in subcortical areas related to cognitive control such as the dorsal striatum (Laube et al., 2020), while others do not (Alarcón et al., 2014; Peters et al., 2013). However, longitudinal studies can help more fully understand how puberty onset impacts plasticity, as they focus on how changes in brain structure and function over time are related to pubertal hormones.

To date, only a handful of longitudinal studies have investigated changes in cortical and subcortical networks together with pubertal changes in gonadal hormones (for a detailed overview on both cross-sectional and longitudinal literature, see Dai and Scherf, 2019; Vijayakumar et al., 2018). Changes in pubertal testosterone associated with pubertal onset have been related to grey matter volume changes in subcortical structures including the amygdala, hippocampus, and striatum (Goddings et al., 2014; Herting et al., 2014; Wierenga et al., 2018), albeit with different directions depending on sex. For instance, Herting et al. (2014) showed that changes in testosterone levels during early puberty were associated with decreases in amygdala volume for boys, but with increases for girls. In addition, changes in cortical and subcortical grey and white matter volume overall, as well as specifically in the striatum and amygdala, were larger during early puberty than during late puberty. These results suggest that subcortical networks are sensitive to rises in pubertal hormones such that changes within these networks are more pronounced during early than late puberty (Herting et al., 2014). Similarly, Brouwer et al., 2015 found no associations between female gonadal hormones and grey matter development in 9-year old twins. However, three years later, at age 12, estradiol levels were negatively correlated with grey matter density in the left frontal and parietal regions. Finally, a study by Nguyen et al. (2013) scanned participants ranging from 4 to 22 years every 2 years and found that increases in testosterone predicted decreases in cortical thickness in posterior cingulate and dorsolateral prefrontal cortex (DLPFC) for post-pubertal boys. In contrast, pre-pubertal girls showed a positive relationship between testosterone change and volume change in the somatosensory cortex, such that increases in testosterone predicted increases in somatosensory thickness. This pattern was reversed for post-pubertal girls: Here, testosterone was related to a decrease in thickness of the somatosensory cortex. These results suggest that the effects of testosterone on grey matter in the somatosensory cortex differ depending on its level, as well as on the individual’s sex. In other words, the findings of Nguyen et al. (2013) can be interpreted as suggesting the possibility of different effects of executive function training in boys and girls, moderated by pubertal onset.

In summary, these selective examples highlight that hormones show distinct effects on brain development before and after pubertal onset. Depending on the timepoint at which hormones are measured during development, they have been related to both increases or decreases in brain volume with clear differences between sexes and brain regions. Overall, evidence shows that structural changes are more pronounced during early than late puberty, suggesting that the rate of change in hormone levels may be an important factor for brain development.

As testosterone or estradiol levels during childhood are lower than during adolescence, one could assume that pre- and post-pubertal timing effects may not only depend on changes in hormones but also on individual differences in levels of hormones. In accordance with this, levels of testosterone were found to moderate the covariance between the amygdala and the medial prefrontal cortex (mPFC) in a longitudinal sample aged between 6 and 22 years (Nguyen et al., 2015). More specifically, individuals with lower testosterone levels had larger amygdala volumes and greater cortical thickness in mPFC, whereas those with higher levels had smaller amygdala volumes and less mPFC thickness. In contrast, testosterone had the opposite effect on cortico-hippocampus covariance: Here, higher testosterone levels were related to larger volumes in the hippocampus and greater whole-brain cortical thickness, whereas lower levels were related to lower volume of the hippocampus and less cortical thickness. Yet, the modulatory effect of testosterone on cortico-hippocampus covariance was only apparent in boys (Nguyen et al., 2017). Interestingly, higher testosterone levels, which were related to larger hippocampus volumes and whole-brain cortical thickness, were also associated with lower performance on specific components of executive function, such as monitoring and shifting between actions. This study is, to our knowledge, the first to combine pubertal hormones, structural brain measures, and behavioral correlates of executive functioning. It suggests that pubertal testosterone may decrease capacity for learning, as higher levels correlated with lower executive functioning. Thus, interventions aiming at training processes based on hippocampal functioning may show differential effects depending on the onset of puberty and levels of testosterone, especially in boys.

Regarding changes in white matter development, we are not aware of any longitudinal studies that have included measures of pubertal hormones. One study by Herting and Sowell (2017) related pubertal changes in physical development as measured by the Pubertal Developmental Scale (PDS) to changes in fractional anisotropy (FA) at two timepoints two years apart. They found sex-specific changes in FA, where boys showed increases in the superior frontal gyrus and precentral gyrus, and girls showed decreases in the anterior corona radiata.

Only a handful of longitudinal studies have investigated how changes in the blood-oxygen-level-dependent (BOLD) signal in magnetic resonance imaging (MRI) are related to increases in pubertal hormones across adolescence. For instance, Braams et al. (2015) showed that increased activation in the nucleus accumbens for wins over losses in a heads-or-tails gambling task scaled linearly with testosterone in a longitudinal sample of individuals between 8 and 27 years of age. As the nucleus accumbens is implicated in reward processing (McClure et al., 2004; Schultz et al., 1992), this suggests (although from a reversed-inference perspective) that training benefits, for example, may be especially moderated by rewards after pubertal onset, as well as by emotional context (see Laube and van den Bos, 2016). Similarly, Spielberg et al. (2014) found that increases in testosterone levels over two years starting at 11–12 years in girls and 12–13 years in boys predicted increased responses in the amygdala and nucleus accumbens to fearful faces two years later. In a follow-up study, Spielberg et al. (2015) found that testosterone increases over time were associated with decreased connectivity between the amygdala and the orbitofrontal cortex when processing fearful faces, suggesting that increased amygdala activation is related to a functional decoupling between the amygdala and the orbitofrontal cortex due to increases in testosterone. Since the amygdala plays a prominent role in fear processing (LeDoux, 2003) this suggests that hormonal changes at puberty onset may represent one potential factor contributing to risk for anxiety-related disorders (Reardonet al., 2009).

Regarding testosterone’s influence on prefrontal control networks, we recently showed, albeit only cross-sectionally, that when adolescents made more patient choices in an intertemporal choice paradigm, pubertal testosterone was positively correlated with activity in those parts of the dorsal striatum that mainly project to the PFC (Laube et al., 2020). That is, individuals with higher testosterone showed more activity in the dorsal striatum when choosing the more patient, larger later option compared to individuals with lower testosterone levels, indicating that testosterone may modulate the recruitment of top-down control for choosing the more patient choice.

With respect to episodic memory, a recent study examining 8–14-year-olds on up to three measurement occasions found that pubertal development was associated with longitudinal changes in hippocampal and frontal activity during memory retrieval (Selmeczy et al., 2019). Highlighting the importance of pubertal timing, this study demonstrated that increases in testosterone were associated with increased fronto-hippocampal task activation for children who were older at the initial assessment. At the same time, initially younger children showed decreases in fronto-hippocampal activation over time.

On the one hand, increases in pubertal hormones are consistently related to increased activation within specific subcortical regions such as the amygdala, nucleus accumbens, and dorsal striatum. On the other hand, increases in pubertal hormones also coincide with decreased functional connectivity between subcortical and cortical networks, particularly pronounced at early pubertal timing. Consistent with the patterns found in animal research, the human neuroimaging literature points towards a coherent pattern of pubertal hormones impacting grey matter and functional activity changes in subcortical and cortical regions. As decreases in grey matter are related to cell loss and synaptic pruning (Kolb and Gibb, 2011), puberty may also play a pivotal role in triggering neuronal pruning, which typically occurs during adolescence. For instance, the study by Hebbard et al. (2003) found that pubertal testosterone was related to LTP in the hippocampus in male rats, which has been identified as an important factor for the elimination of synaptic contacts (see Selemon, 2013). This is further supported by the finding of Liu et al. (2012) showing that BDNF signaling regulated by sex hormones directed pruning of sensory axons in the mammary gland. However, at present, it is difficult to answer whether increased pruning during adolescence signifies more or less potential for change.

To summarize, structural and functional brain changes during adolescence have been related to the timing and level of pubertal hormones. The existing findings indicate that puberty-related structural change may entail increases or decreases in gray and white matter volume, and that pubertal effects may vary between sexes, brain regions and according to the timing of puberty. How this puberty-related structural change impacts higher cognitive functioning and improvements with training is yet to be explored, as learning-related changes have not been tested extensively so far. While longitudinal data points towards consistent increases in the activation of subcortical regions such as the amygdala and nucleus accumbens that are related to increases in pubertal testosterone, testosterone effects on the PFC and learning have, to our knowledge, not been tested so far. Yet with regard to the cognitive training literature reviewed before, the fronto-parietal network which is involved in episodic and working memory, relational reasoning, and task-switching shows consistent structural changes sensitive to puberty onset (Nguyen et al., 2013, 2015, 2017).

## 8 General summary & conclusions

Is adolescence a period of increased or decreased plasticity for higher order cognition? Evidence suggests a rather nuanced view of the plasticity-related changes in adolescence, where an increase or decrease depends on the respective domain of higher-order cognition, brain regions, and the individual’s sex. Specifically, our review of age-comparative cognitive training studies revealed both increases and decreases in training-related benefits, depending on the cognitive domain, the type of training, and the age group being tested (see Table 1). Given that none of the training studies reviewed here included measures of pubertal development, the mixed evidence across those studies may also be due to different pubertal stages of participants that were included in the same age groups. For instance, Knoll et al. (2016) created three adolescent groups based on three bins of equal size that do not necessarily coincide with specific biological changes. Thus, it is possible that some individuals in their early adolescent group were not yet in puberty and thus, in biological terms, still children, as pubertal onset represents a clear biological marker of the beginning of adolescence. Pubertal onset has been shown to have high inter-individual variability (Lee, 1980). This heterogeneity in entry into puberty may also contribute to some of the other existing findings. For example, Brehmer et al. (2007) reported substantial interindividual differences in episodic memory plasticity particularly in the younger children (9–10 years), which may be related to individual differences in pubertal onset.

Turning towards the effects of pubertal onset on the mechanisms of plasticity, the picture becomes more coherent. Animal studies investigating single-cell mechanisms implicated in the opening or closing of sensitive periods indeed strongly suggest that pubertal hormones impact adolescent plasticity. Overall, it seems probable that pubertal hormones impact PV and BDNF expression, as well as GABA neurotransmission in a region-specific manner, particularly in the hippocampus and the frontal cortex. If learning was tested in these animal studies, pubertal hormones were mostly related to decreases in performance, suggesting decreased learning. These findings are echoed in a recent human imaging study showing that higher testosterone levels across puberty were related to larger hippocampal volumes and higher overall cortical thickness, which reduced executive functioning performance over time (Nguyen et al., 2017). Yet, these conclusions are preliminary in nature, and human imaging studies are needed to assess behavioral changes in executive functioning and episodic memory in relation to hormonal measurements.

## 9 Roadmap to study plasticity-related changes in adolescence

How should we proceed in investigating adolescent plasticity for higher-order cognition? Rather than generalizing across the brain, the direction of plasticity-related changes in adolescence may very well depend on the specific cognitive domain and brain networks being studied.

### 9.1 Reliable measures of plasticity-related changes

A clear definition of plasticity is necessary, as this sets boundaries on what type of studies we need to do. Particularly, if we want to draw conclusions about the potential increase or decrease of brain malleability during adolescence, we need to reliably assess benefits from a cognitive training intervention over time, ideally accompanied by measures of brain structure and function to evaluate whether the range of available resources has changed. The application of longitudinal training designs, where a specific cognitive skill is trained and improvements are assessed over time, is essential along with repeated hormone measurements. One important characteristic of the training has to be adequate challenge, or ensuring a mismatch between the available resources and environmental demands, so that every participant has room for improvement. This is particularly important when comparing different age groups, as children usually show lower baseline performance in cognitive tasks compared to adolescents, and thus have more room for improvement. As a consequence, without ensuring comparable challenge across age groups, higher benefits would represent an artefact of task difficulty and the way performance was measured. In order to overcome this issue, adaptive training designs can be used which individually guarantee similar challenges for all participants. Moreover, integrating both active and passive control groups within training designs would be crucial in order to tease apart normative versus experience-dependent changes.

In addition, we need reliable measures to capture training-related improvements. Here, using multiple measures per domain (Schmiedek et al., 2010) can be particularly helpful to ensure that changes in underlying ability can be assessed reliably. Furthermore, as brain changes during development are diverse and multifaceted, we also suggest to utilize measures that are sensitive to change in different aspects of brain structure and function. As our review of the literature revealed, findings may vary based on whether task-related activity or connectivity are measured (Spielberg et al., 2015, 2014) and we argue that combining various MRI modalities in a hypothesis-driven way can provide helpful insights into the effects of pubertal hormones on plasticity. The surge of new methodological developments (e.g., Marques et al., 2010; Weiskopf et al., 2013) is particularly favorable regarding improved measurement of specific training-related changes in brain structure. In addition, magnetic resonance spectroscopy is a promising method that could be integrated in cognitive training studies to shed more light on the impact of gonadal hormones on mechanisms contributing to adolescent plasticity, such as GABA.

### 9.2 Reliable measures of puberty

The question *if* and *for what* adolescence is a phase of increased or decreased plasticity can only be fully answered if there is also an answer to the *how*. We argue that assessing measures of pubertal development is crucial to resolve mixed evidence regarding plasticity in adolescence and to better understand individual differences in potential for change. Specifically, animal research points to pubertal onset as one crucial factor in modulating plasticity in adolescence (Piekarski et al., 2017a, b). As the main marker of pubertal onset is an increase in levels of pubertal hormones, the *increase* in gonadal hormones over time has to be assessed and used as a predictor for training-related gains. Here, latent change score models (Kievit et al., 2018) can be used to statistically model changes in hormones levels and examine how they are related to changes in brain and behavior. Ideally, participants in a training study can be chosen based on their pubertal status, so that training-related benefits can be also compared between pre-pubertal children and pubertal adolescents. This is particularly important given that there is sizeable interindividual variability in pubertal onset, which is not captured by chronological age. Yet, to systematically compare pre- and post-pubertal individuals, a reliable measure of pubertal onset is necessary. This may be achieved by combining different methods of hormonal change measures, for instance via hair and saliva, along with self-report measures of external physical changes assessed via the PDS or Tanner staging. To conclude, puberty-comparative (rather than age-comparative) cognitive training studies may be a fruitful way to shed more light on the question whether adolescence is a period of increased and/or decreased plasticity for higher cognitive functions, and how.

### 9.3 Consideration of social context and function of pubertal development

Along with the questions if, for what and how adolescence represents a sensitive phase for higher-order cognition, the question of the respective context may be crucial as a potential moderator of the relationship between pubertal hormones and plasticity. For instance, studies in adults have shown that the behavioral effects of testosterone are only present in a context where social status is threatened (Sapolsky, 1991). In particular, if social status is challenged, any type of behavior is enhanced by testosterone to regain higher social status (Eisenegger et al., 2010). Thus, future research should also investigate how changes in the (social) environment due to factors like an individual’s physical changes after pubertal onset may impact mechanisms of plasticity and learning. In addition, pubertal hormones play an important role in developmental changes in social motivational processes, leading to an increase in socially motivated behavior (Cardoos et al., 2017; Crone and Dahl, 2012). This increased motivation for social goals may also facilitate learning in higher cognition. Importantly, it has been hypothesized that hormones may play a role in setting of a sensitive period for sociocultural processes (Blakemore and Mills, 2014), suggesting that plasticity may be increased specifically for learning about the social environment. Indeed, animal work has also suggested that the hormonal processes associated with sexual differentiation that happen around puberty facilitate the behavioral changes that are needed to adapt behavior to new roles (for a review see Adkins-Regan, 2007). Future studies would benefit from linking changes in pubertal hormones to plasticity in social brain functions.

Related to the adaptive function of pubertal hormones, life history theory puts an emphasis on reproductive success and highlights the coordinating effects of hormones (Roney, 2016). Specifically, androgens are thought to manage tradeoffs in the investment of finite resources across the life cycle, with the overall goal to promote lifetime reproductive success. A significant increase in pubertal hormones at the onset of puberty clearly shifts an organism’s energy allocation from long-term, survival-related activities, to short-term mating-related behaviors. Since plasticity is accompanied by high metabolic costs and occurs only in the event of a prolonged mismatch between current resources and environmental demands, plasticity should thus generally decrease with increasing age. Yet, for successful reproduction, social skills are beneficial to win a potential mating partner, so the onset of puberty may also characterize a temporary offset of decreasing plasticity in the socioemotional domain, or even an increase. If so, a tempting hypothesis to be tested in future research is that plasticity in cognitive tasks related to hormonal changes may be boosted by plasticity mechanisms that may have been initially focused on social behavior. Taken together, a holistic view integrating both the socioemotional and cognitive domain together with its interactions may be a key to fully understand and characterize adolescent plasticity and learning.

### 9.4 Practical applications that follow from the interaction between pubertal hormones and plasticity

Certainly, while these types of studies require a sizeable amount of resources, the gained insights would have wide implications for educational and clinical settings. If pubertal onset indeed diminishes plasticity for specific higher-order functions, this would suggest that, for example, second or third languages should be taught earlier. Moreover, given a possibility to ameliorate developmental deficits through training (see Jolles and Crone, 2012), pubertal onset may be a critical factor that should be taken into account when deciding about the timing of psychological interventions. Relatedly, as pubertal onset occurs earlier in girls than in boys (Dorn et al., 2006), students’ sex may also be a relevant factor for educational and psychological interventions. While none of the reviewed cognitive training studies assessed sex differences in training benefits, animal and human imaging studies point towards sex-specific effects for plasticity (Herting and Sowell, 2017; Piekarski et al., 2017a, b). Future research should therefore also test sex differences in benefits through cognitive interventions in studies that select their participants solely on the basis of age. Due to sex differences in pubertal timing, we would expect girls to benefit less than boys if pubertal onset decreases plasticity for higher cognitive functioning. Of note, we argue here that these effects should not be seen uniformly across adolescence, but should be specific for the period around puberty onset, or early adolescence. With adolescence extending into the early 20s, future research is needed to tackle potential changes in executive functioning and episodic memory plasticity with the transition from adolescence into adulthood, and the underlying neural mechanisms.

Practical implications that follow highlight the importance of tailoring learning objectives and approaches to pubertal status instead of just age in a school environment individually. In addition, adolescence is often associated with increases in anxious and depressive symptoms, especially in females (McLaughlin and King, 2014). Treatments for these symptoms that rely on improving higher-order cognitive abilities, such as the attention-bias modification training (Mogg and Bradley, 2018) could benefit from a better understanding of the influence of pubertal onset and hormones on attention in order to tailor treatment to the individual adolescent.

### 9.5 Investigating the effects of pubertal timing on plasticity and learning

As pubertal onset has moved forward to younger ages throughout the last decades (Aksglaede et al., 2008), understanding the impact of early puberty on learning and plasticity is crucial and urgent. An early entry to puberty is associated with a variety of mental health problems, such as depression, anxiety, eating and conduct disorders or schizophrenia, as well as substance use and lower academic achievement (Mendle and Ferrero, 2012; Mendle et al., 2010, 2007; Mensah et al., 2013; Walvoord, 2010). Based on evidence demonstrating an impact of pubertal hormones on regulating sensitive periods of cognitive development, an early entry may close sensitive periods for cognition too soon, before basic cognitive functions are fully developed. This may introduce larger individual differences in available neural resources after pubertal onset, which in turn changes the starting point for plasticity during adolescence.

Even if pubertal hormones have primarily focal effects on specific brain regions, these regions are embedded in broader neural networks. An earlier decrease in plasticity in specific regions due to early puberty onset could thus disturb the balance within an entire network, potentially having long lasting negative effects on learning and development (but see Chaku and Hoyt, 2019). Based on the evidence reviewed above, we suggest that fronto-hippocampal and fronto-striatal networks may be particularly susceptible to early puberty onset. Besides pubertal onset, adolescents show large variations in the duration of and progression through puberty (e.g., Marceau et al., 2012; Marshall and Tanner, 1970). However, to our knowledge, only one study has directly tested a relationship between pubertal tempo and cognitive development, showing no significant associations with executive functions such as attention and self-control (Chaku and Hoyt, 2019).

Excitingly, recent research offers a perspective for the reopening of sensitive periods. For instance, attention training can reinstate plasticity later in life (Werker and Hensch, 2015). If we know exactly which functions are sensitive to pubertal hormones, and can identify children with a particularly early onset of puberty, specific training regimes could be directly targeted to prevent or limit the potential negative effects of early puberty. In general, understanding which cognitive functions can be particularly well trained either before or after puberty and understanding the reasons for such increased or decreased plasticity represents an important next step for future research.

## Declaration of Competing Interest

The authors declare no conflict of interest.
